# A retrospective cross-sectional study to determine chirality status of registered medicines in Tanzania

**DOI:** 10.1038/s41598-020-74932-x

**Published:** 2020-10-20

**Authors:** Kissa W. Mwamwitwa, Raphael M. Kaibere, Adam M. Fimbo, Wilber Sabitii, Nyanda E. Ntinginya, Blandina T. Mmbaga, Danstan H. Shewiyo, Morven C. Shearer, Andrew D. Smith, Eliangiringa A. Kaale

**Affiliations:** 1 Pharm R&D Lab and Department of Medicinal Chemistry, School of Pharmacy, Muhimbili University of Health and Allied Sciences, P. O. Box 65545, Dar es Salaam, Tanzania; 2 Tanzania Medicines and Medical Devices Authority, P. O. Box 77150, Dar es Salaam, Tanzania; 3 School of Medicine, University of St, Andrews, Fife, KY16 9TF Scotland, UK; 4 National Institute of Medical Research - Mbeya Medical Research Centre, P. O. Box 2410, Mbeya, Tanzania; 5 Kilimanjaro Clinical Research Institute, P. O. Box 2236, Moshi, Kilimanjaro Tanzania; 6 Kilimanjaro Christian Medical University College, P. O. Box 3010, Moshi, Kilimanjaro Tanzania; 7 School of Chemistry, University of St, Andrews, Fife, KY16 9TF Scotland, UK

**Keywords:** Drug discovery, Medical research, Chemistry

## Abstract

Medicines with a stereogenic center (asymmetric carbon) are mainly present as racemates with a mixture of equal amounts of enantiomers. One enantiomer may be active while the other inactive, alternatively one may produce side-effects and even toxicity. However, there is lack of information on the chirality status (either racemates, single active enantiomer or achiral) of medicines circulated on the market particularly in African countries. We established the chirality status of registered medicines in Tanzania by conducting a retrospective cross-sectional study. Registration data for the past 15 years from 2003 to 2018 were extracted from TMDA-IMIS database to Microsoft excel for review and analysis. A total of 3,573 human medicines had valid registration. Out of which 2,150 (60%) were chiral and 1,423 (40%) achiral. Out of the chiral medicines, 1,591 (74%) and 559 (26%) were racemates and single active enantiomers, respectively. The proportion of racemates within chiral medicines was considerably higher than single enantiomer medicines. The use of racemates may cause harm to the public and may contribute to antimicrobial resistance due to potential existence of inactive and toxic enantiomers. In order to protect public health, regulatory bodies need to strengthen control of chiral medicines by conducting analysis of enantiomeric impurity.

## Introduction

Chirality is very important in the pharmaceutical field^1–3^. Chirality (sometimes called stereoisomerism, enantiomerism or dissymmetry) is a property of an object which renders it non-superimposable with its mirror image^1–7^. Chiral medicines are those medicines with a stereogenic center (often called an asymmetric carbon) and exhibit chiral properties^1–4^. Most pharmacological processes present a high degree of stereoselectivity which results in differences between the activities of the enantiomeric forms of chiral medicines^1,4,8,9^. It is well known that a racemic mixture consists of an equimolar mixture of two enantiomers of the same chemical structure^8^. Enantiomers in some chiral medicines may exhibit marked differences in biological activities such as pharmacology, toxicology and pharmacokinetics^1,8,10,11^. One enantiomer may be active while the other inactive, and may produce side-effects and/or exhibit toxicity^6,8,12,13^. The use of racemic mixtures may present problems, such as adverse effects or toxicities particularly if they are associated with a less active, or inactive isomer^8,14,15^.

**Figure 1:**
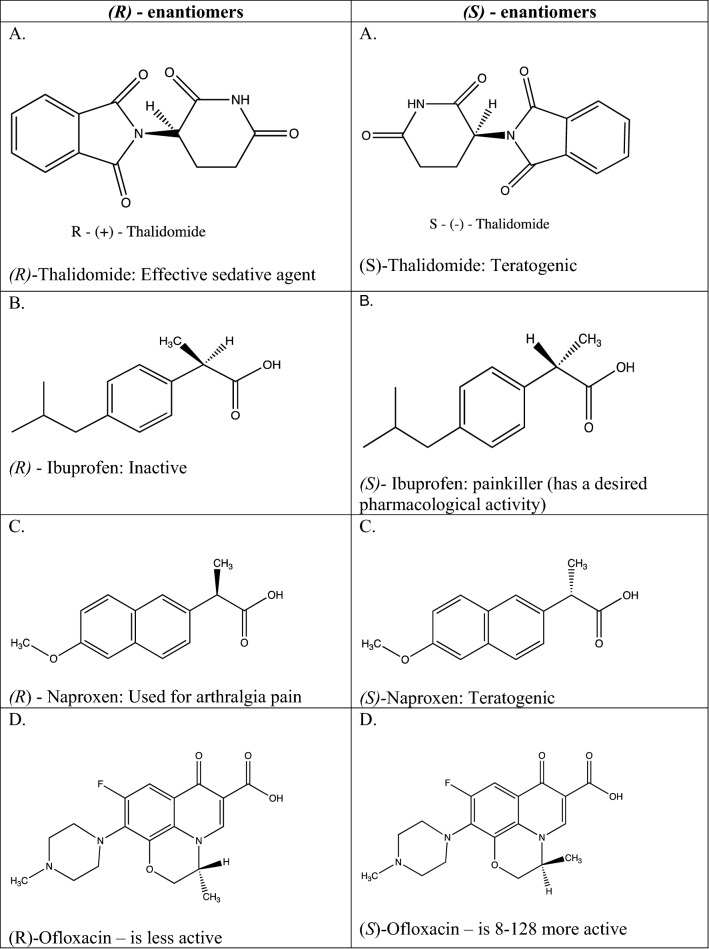
Examples of structures of *(R)*- enantiomers: A—D and *(S)*- enantiomers: A—D of chiral medicines and their activities.

There are many examples of chiral medicines whose enantiomers vary drastically in their properties. A well-known example of enantiomer related toxicity is the (*R)-* and *(S)-*enantiomers of thalidomide^1,2,4,8^. The (*R*)-enantiomer of thalidomide is an effective sedative agent while, the (*S*)-enantiomer is known to cause teratogenic birth defects^1,4,8^. These defects included phocomelia, a severe shortening or lack of limb structures^8^. Ibuprofen, a painkiller has (*S*)-enantiomer with desired pharmacological activity while the (*R*)-enantiomer is totally inactive^1,3,16^. (*R*)-Naproxen is used for arthralgia pain while (*S*)-Naproxen is teratogenic^2^. Ofloxacin has a chiral mixture of levofloxacin [(*S*)-Ofloxacin] and dextrofloxacin [(*R*)-Ofloxacin], in which levofloxacin is 8–128 more active than dextrofloxacin^17^. So far, many chiral medicines are still used as racemates. Examples of structures of *(R)-* and *(S)-* enantiomers of chiral medicines and their activities have been shown in Fig. 1.

Worldwide, there is no mandatory regulatory requirement to enforce the development of new medicines exclusively as pure active single enantiomers^13,18^. Some regulatory agencies leave the decision of a racemate or a single enantiomer formulation of a new medicine to the manufacturers^4,13,19^. However, the choice to make a racemic mixture versus a single enantiomer formulation must be justified based on quality, safety and efficacy together with the risk–benefit ratio between the two forms^4,13,19^. Due to the lack of regulatory requirements and increased technological developments, the number of new chiral entities as a pure active single enantiomers is increasing^5,15^. This contributes to the availability of both single enantiomers and racemic mixtures circulating on the market particularly in developing countries such as Tanzania^5,15^. Nonetheless, it has been reported that some manufacturers are marketing more single enantiomers of the old racemic drugs as a new drug; this is known as chiral switch, and has claims of greater activity, less toxicity or both^1,5^. In addition, chiral switches have also contributed to a number of agents being commercially marketed as both single enantiomer and racemic mixture^4,20^.

Few studies conducted in some developed countries such as United States of America (USA) and Japan revealed that more single enantiomers are approved compared to racemates^18,21^. In the period between 2001 and 2011, the United States Food and Drugs Administration (USFDA) approved registration of 15 single enantiomeric medicines. These medicines showed improved therapeutic index through increased potency, selectivity and decreased side-effects compared to its corresponding racemate^21^.

A review of chiral medicines approved for use in Japan was conducted between 2001 and 2003 and the results indicated an increased trend towards development of single enantiomer medicines^18^. During that period, Japan approved 3 types of chiral medicines that were classified as single enantiomer (48%), racemic (13%) and achiral (39%). It is notable that single enantiomer medicines were produced three times more than racemic medicines^18^.

In developing countries, little attention has been given on the importance of chiral medicines^22^. Some guidelines for submission of technical documents for medicines registration particularly in Africa have included the requirement for submission of evidence of the occurrence of isomers, chirality or polymorphorphism^23–25^. However, there is no requirement for the development of either single enantiomer medicines or racemic mixtures^6,23–25^. The decision regarding the development of a single enantiomer or racemic mixture is left to the manufacturers^24^. Moreover, there is no need for manufacturers to give justification as to why they have decided to formulate single or racemate medicines^23,24^. In addition, National Medicines Regulatory Agencies (NMRAs) in African countries have not set systems to conduct assessment of registered medicines to establish their chirality. Consequently, there is lack of information on the chirality status of registered medicines circulating on the market within the African region including Tanzania.

One of the functions of the Tanzania Medicines and Medical Devices Authority (TMDA) is to register quality, safe and effective medicines^25^. Since its establishment many human medicines have been approved of which some are chiral medicines^26^. However, their chirality status (single enantiomer or racemic mixtures) is not known. It is important to know the chirality status of medicines circulating on the market as such products may cause adverse effects to the users^1,4,7,8,14^. Therefore, this study aimed to establish the chirality status of registered human medicines in Tanzania. Additionally, it was considered valuable to know the pharmacological groups of registered chiral medicines and their availability in the essential medicine list. The findings can provide common understanding of the status of chiral medicines between manufacturers and regulatory authorities. This will facilitate improvement during development of chiral medicines and also the regulatory requirements.

## Results

### Registered chiral medicines

A total of 3,573 human medicines had valid registration. Out of registered human medicines, 2,150 (60%) were chiral and 1,423 (40%) achiral. Of 2,150 registered chiral medicines, 1,591 (74%) were racemates while 559 (26%) single enantiomers. Moreover, out of all 2,150 registered chiral medicines, 550 were fixed-dose combinations of either chiral—chiral 332 (60%) or chiral—achiral 218 (40%).

Medicines circulating on the market were registered by manufacturers from 45 countries. India registered more medicines 1,860 (52%) followed by Kenya 262 (7%), Germany 156 (4%), China 106 (3%), United Kingdom 109 (3%), Cyprus 100 (2.8%), Tanzania mainland 94 (2.6%), France 75 (2%), Pakistan 69 (1.9%), Switzerland 1.8%, Italy 1.8%, Malaysia 1.5%, Egypt and Belgium 1.4%. Other countries contributed less than 1.4% of the registered medicines. Details on chirality of medicines registered from specific countries have been indicated in Table 1.

**Table 1:** Registered chiral medicines in Tanzania mainland by country of origin.

Country	Registered medicines (n)	Registered chiral medicines (n)	Chirality type (n)		Combination (n)		Registered Achiral medicine (n)
			Racemate	Single Enantiomer	Chiral –Chiral	Chiral—Achiral	
India	1,860	1,149	825	324	185	96	711
Kenya	262	137	123	14	8	19	125
Germany	156	91	53	38	10	12	65
UK	109	76	67	9	24	5	33
China	106	90	67	23	12	13	16
Cyprus	100	46	42	4	5	0	54
Tanzania	94	44	38	6	3	21	50
France	75	26	22	4	3	3	49
Pakistan	69	40	27	13	4	2	29
Switzerland	65	34	21	13	5	5	31
Italy	63	40	26	14	3	3	23
Malaysia	54	34	30	4	4	3	20
Egypt	50	26	19	7	6	4	24
Belgium	50	26	22	4	5	3	24
Others < 50	460	291	209	82	55	29	169
Total	3,573	2,150	1,591	559	332	218	1,423

Out of 45 countries, 14 had registered more than 50 human medicines. Results indicated that all countries have registered both chiral and achiral medicines, in which more chiral medicines were registered compared to achiral medicines with exception of three (3) countries. These countries namely Cyprus, Tanzania and France had registered more achiral medicines than chiral medicines. Results further revealed that all countries registered chiral medicines in both racemic and single enantiomeric forms whilst racemic mixtures predominate over single enantiomers (Table 1).

### Anatomical therapeutic chemical (ATC) classification system

In the first level of ATC codes, results indicated that, the number of registered anti-infective human medicines for systemic use was significantly higher 941 (26%) than medicines used for alimentary tract and metabolism 438 (12.3%, *p* < *0.0001*) and cardiovascular system 390 (11%, *p* < *0.0001*). However, the percentage of registered chiral anti-infective (35%) and cardiovascular (12%) medicines were high compared to chiral medicines in alimentary tract and metabolism (7.8%) and other pharmacological groups. High proportion of chiral medicines within pharmacological groups were observed in systemic hormonal preparations (excluding reproductive hormones and insulin) 97.3% (36/37) and anti-infective 80% (753/941). The proportion of registered chiral medicines in each ATC level is indicated in Table 2.

**Table 2:** Proportion of chiral medicines registered in Tanzania by June, 2018 in each ATC level.

Codes	ATC description	Human medicines (n)	Chiral medicine in each level (n)	Proportion of Chiral medicine in each level (%)	Proportion of achiral medicines (%)
A	Alimentary tract and metabolism	438	167	38	62
B	Blood and blood forming organs	146	61	41.8	58.2
C	Cardiovascular system	390	267	68.5	31.5
D	Dermatological drugs	246	139	56.5	43.5
G	Genitourinary system and reproductive hormones	128	82	64	36
H	Systemic hormonal preparations, excluding reproductive hormones and insulin	37	36	97.3	2.7
J	Anti-infective for systemic use	941	753	80	20
L	Antineoplastic and immunomodulating agents	117	64	54.7	45.3
M	Musculoskeletal system	204	53	26	74
N	Nervous system	296	116	39	61
P	Antiparasitic products, insecticides and repellents	200	114	57	43
R	Respiratory system	262	187	71.4	28.6
S	Sensory organs	129	101	78.3	21.7
V	Various ATC structures	39	10	25.6	74.4
	Total	3,573	2,150		

From Table 2 above, total percentage of chiral and achiral medicine in each ATC pharmacological group is 100.

**Figure 2:**
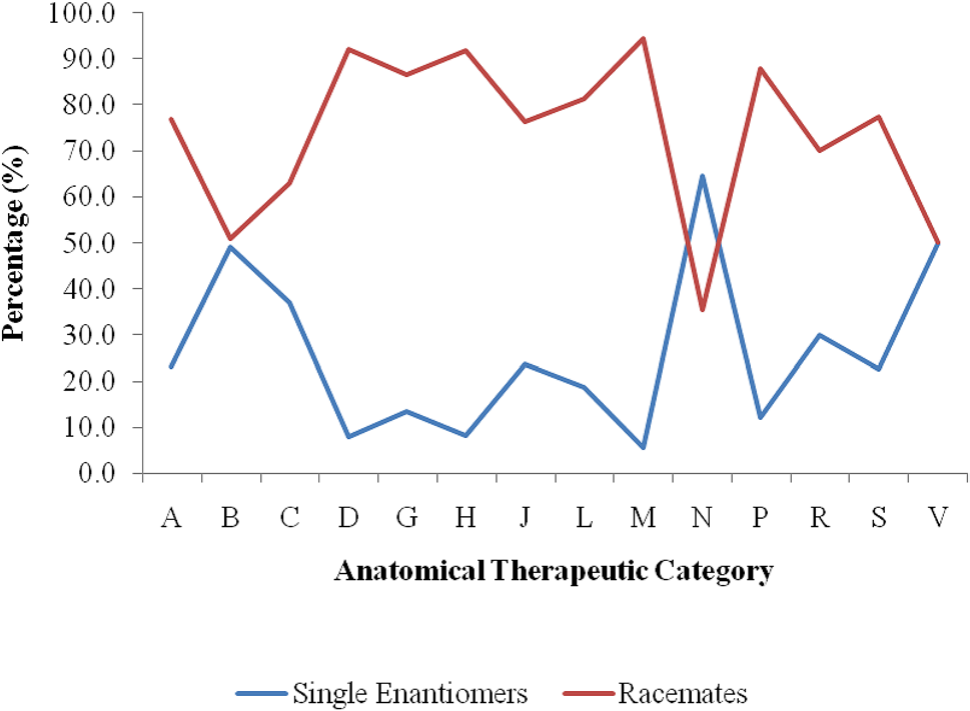
Percentage of Level 1 ATC classification of chiral medicines; racemates is high in all groups except nervous system (N) and various (V). The codes and ATC description are as indicated in the 1st and 2nd column of Table 2.

In each of the 14 main ATC groups, both racemates and single enantiomers were registered. However, more racemates were registered compared to single enantiomers with the exception of various ATC level (V) which had equal number (50%) of racemate and single enantiomer chiral medicines. Only nervous system (N) had more single enantiomers (64.7%) compared to racemates (35.3%) as indicated in Fig. 2.

**Figure 3:**
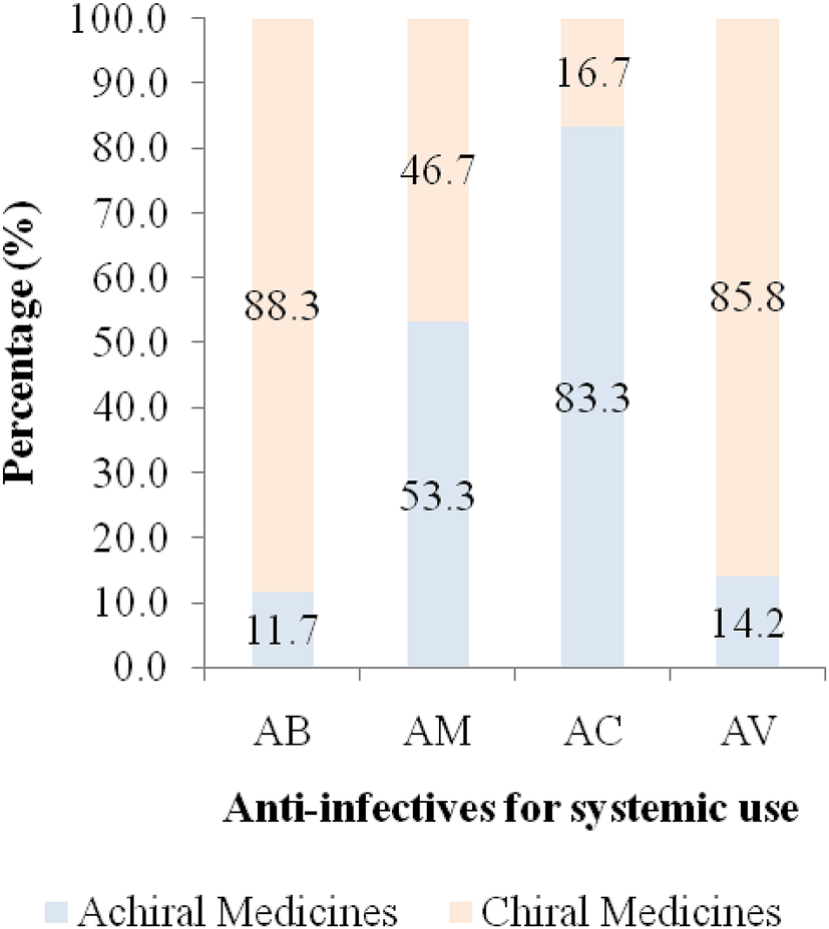
Percentage of chiral medicines in level 2 ATC classification. Key: AB—Antibacterials for systemic use, AM—Anti-mycobacterials for systemic use, AC—Antimycotics for systemic use, AV—Antivirals for systemic use.

**Figure 4:**
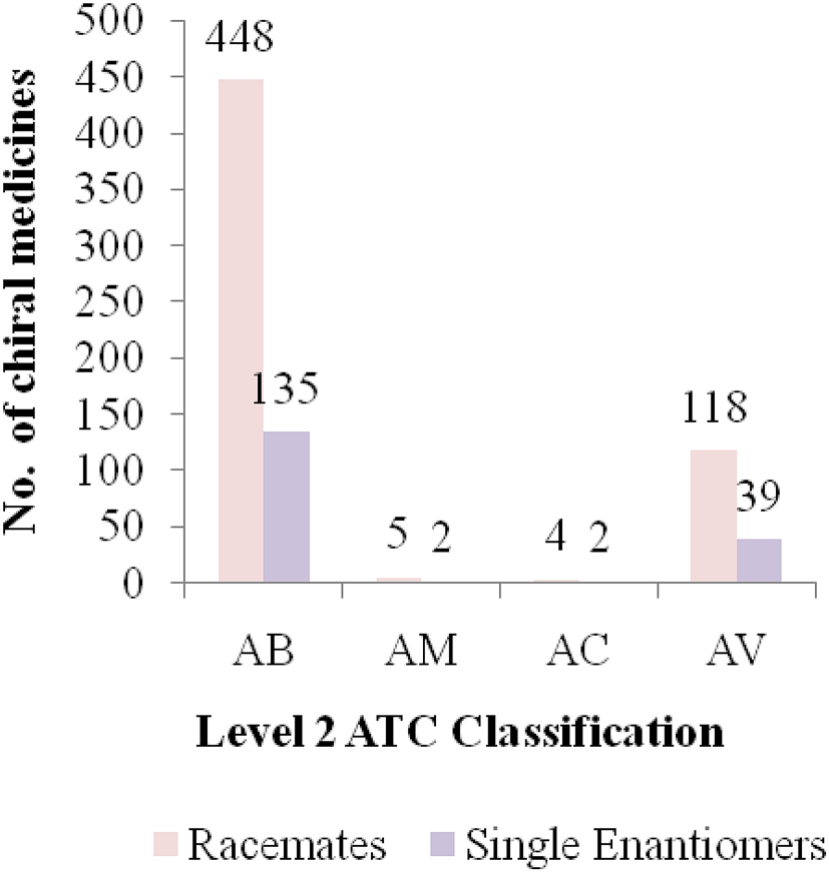
Number of racemate and single enantiomer medicines in each pharmacological group in level 2 ATC classification. Key: AB—Antibacterials for systemic use, AM—Anti-mycobacterials for systemic use, AC—Antimycotics for systemic use, AV—Antivirals for systemic use.

Chiral anti-infective medicines which have been registered in the Tanzania as per level 2 ATC classification were antibacterials for systemic use, antimycobacterials, antimycotics and antivirals for systemic use as indicated in Fig. 3. More chiral medicines had been registered in pharmacological group of antibacterials and antivirals for systemic use compared to other pharmacological groups. All chiral medicines in each pharmacological group had high percentage of racemates compared to single enantiomer as indicated in Fig. 4.

**Figure 5:**
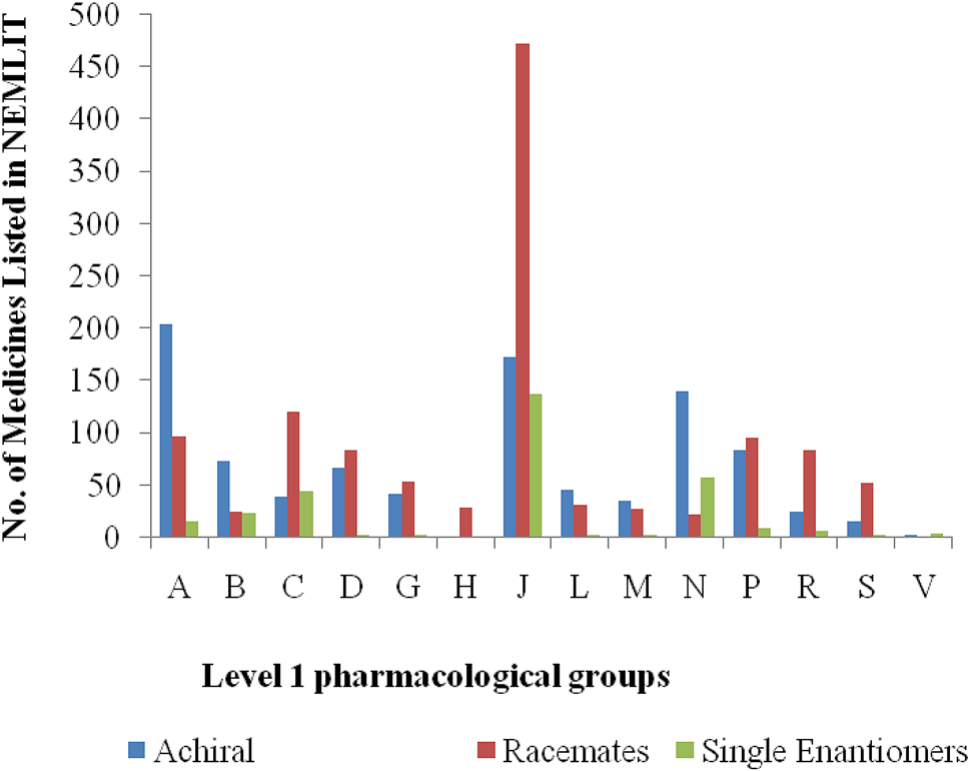
Pharmacological groups of medicines listed in NEMLIT. The codes and ATC description are as indicated in 1st and 2nd column of Table 2.

### Chiral medicines listed in the National Essential Medicines List in Tanzania (NEMLIT)

Out of 3,573 human medicines registered by TMDA, 2,450 (68.6%) were listed in the NEMLIT in which 1,507 (61.5%) were chiral medicines; 1,197 (79%) racemates and 310 (21%) single enantiomers. As per ATC code classification, number of anti-infective medicines for systemic use listed was 782 (32%) which was more as compared to other pharmacological groups as indicated in Fig. 5. Out of 1,123 (31.4%) medicines which were not in the NEMLIT, 481 were achiral and 642 chiral of which 394 were racemates and 248 single enantiomers.

### Most commonly used chiral medicines in Tanzania

The importation records for three years (from 2015/16—2017/18) indicated that Amoxicillin capsules and powder for suspension were the most imported chiral medicines. This means Amoxicillin is highly consumed in Tanzania compared to other chiral medicines. Ceftriaxone injection which is also a chiral medicine was among the top ten imported medicines. These medicines were among the reported medicines with some adverse drug reactions as per Uppsala Monitoring Centre (UMC) Vigiflow/VigiLyze database. The top ten (10) imported chiral medicines for human use are indicated in Table 3.

**Table 3:** Top ten most commonly used chiral medicines for past three years (2015/16—2017/18).

No	Common name
1	Amoxicillin capsule, powder for suspension
2	Phenoxymethylpenicillin tablets
3	Benzyl Penicillin powder for injection
4	Artemether + Lumefantrine tablets
5	Gentamicin injection
6	Artesunate powder for injection
7	Ampicillin Powder for injection
8	Tenofovir disoproxil, Lamivudine, and Efavirenz tablets
9	Cefalexin capsule
10	Ceftriaxone powder for injection

## Discussion

In this study, we conducted retrospective assessment of all registered human medicines to establish chirality status by determining ratio and types of chiral medicines circulating on the Tanzanian market. The results revealed existence of high percentage of registered chiral medicines (60%) with both racemates (45%) and single enantiomers medicines (15%) available. Within the classification of chiral medicines, racemates (74%) predominated over single enantiomer medicines (26%). These findings contrast most of studies that indicate an increased number of single enantiomer drugs and only 25% to 40% of medicines are used as racemic medicines^7,27^. A review on chirality done in Japan^17^ and in United Kingdom (UK)^28^ reported the tendency of registration approval of medicines has been observed to be towards chiral switching or development of the pure enantiomer^28^. The trend indicated development of 60% single enantiomers with only 5–10% of racemic mixtures and 30–35% achiral medicines^17^. A study conducted in Argentina on the pharmacology of chiral 2-arylpropionic acid derivatives indicated about a quarter of all therapeutic agents were marketed and administered as racemic mixtures^29^ while the other findings for a study on drug chirality in anesthesia stated one-third of all synthetic drugs are chiral and marketed as racemates^30^. Some studies indicated that more than half of the medicines were chiral; however, single enantiomers were more registered than racemates^31^. On the other hand, a study conducted on side effects and toxic reactions of chiral drugs^32^ and a review study on chiral drugs conducted in 2006 reported more than half of the medicines on the market were chiral with more approved racemates^1,32^. These earlier studies are in-line with our findings in which more than 50% of the medicines were chiral with more racemates registered and marketed for the past 15 years compared to single enantiomers. It has been reported that regulatory authorities in countries like U.S, Canada, Europe, China and Japan are emphasizing only on active enantiomers of chiral medicines be brought into the market. They have also issued guidelines to manufacturers outlining this requirements^19,33^. This means that the trend towards development of single enantiomers and their use depends on how stringent the regulatory authority is, and the existence of guidelines or requirements on registration of such medicines.

Moreover, our results have revealed high percentage (60%) of registered chiral medicines formulated as fixed-dose combinations of chiral—chiral medicines either with racemates or single enantiomers or both, and the remaining 40% as chiral—achiral combinations. The use of the fixed-dose combination containing racemic mixtures can create more risk to patients as enantiomers may have different pharmacological activities and different levels of toxicity^11^. There are many examples of either fixed-dose combination or co-administration of chiral medicines with reported adverse events^34^. A study conducted in UK on chirality and its importance reported that senior medical advisors on regulatory bodies emphasized on the use of single chemical entities rather than combination of medicines in safeguarding the patient^11^. It has been reported that single enantiomers have less complex and more selective pharmacodynamic profiles and hence, have lesser adverse drug reactions, improved therapeutic profile and have less chances of drug interactions compared to racemic mixtures^2^. Therefore, in order to protect the public from any harm caused by the use of racemic medicines, regulatory bodies should take measures to strengthen the control of chiral medicines even for those existing in a fixed-dose combination and clear guidelines must be laid down^11^.

Our results further indicate low percentage of registered single enantiomers against racemates compared to developed countries such as USA, EU and Japan where the trend of approving single enantiomers is increasing. The increased approval of single enantiomers in developed countries is contributed by existence of chiral separation technology^17,19,20^, the technology which is either minimal or lacking in developing countries. The results also indicate that manufacturers from European countries (such as Germany, UK and France) have been registering more racemic chiral medicines in Tanzania compared to single enantiomers. This was observed within antiretroviral and some of antihypertensive medicines. The reasons for registering low number of single enantiomers could be a result of non-existence of regulatory requirements to compel manufacturers to study each enantiomer and provide justification onto why a single enantiomers or racemates are beneficial^23^. The cheaper price of racemic active pharmaceutical ingredients (APIs) compared to single enantiomers may also explain their higher representation among registered medicines^21,29^. It has been reported in some studies that some API manufacturers import cheaper crude racemic mixtures of chiral medicines^21^ which are expensive to separate single enantiomers on a large scale^30,35^ and leaving the racemic mixture in the final drug product. An additional reason is that, due to lack of capacity in terms of skills, knowledge and infrastructure within regulatory bodies to conduct enantiomeric purity separation during post marketing surveillance studies and therefore, trigger manufacturers to register and market more racemic chiral medicines.

These results support some studies that indicated, necessity of promoting and conducting chiral separation of these medicines especially during quality control stage as chirality plays a key role in clinical therapeutics^1^. It has also been reported that, the existence of problems caused by lack of techniques for separation of chiral enantiomers in medicines needs stricter control by regulatory authorities and detailed consideration during approval of newly-developed medicines^7,18,36^. Most regulatory authorities in developed countries have set regulatory controls for chiral medicines. For example, the USFDA released a policy statement on the development of stereo-isomeric medicines in May 1992^20,33,37^. The policy requires properties of each enantiomer be studied separately before decisions are made to market the medicines as one of the enantiomers or as a racemate^13,14,38^.

Results also indicate that the majority of chiral medicines were registered by Indian companies amounting to 52% of all registered chiral medicines in which 44% were racemic mixtures. This indicates that chiral medicines circulating in Tanzania are mainly imported from India. These results are also consistent with number of imported medicines in Tanzania where by 47% of them come from India^39,40^.

Most chiral medicines registered in Tanzania are classified in the pharmacological group of anti-infectives (35%). In addition, results revealed that 32% of all medicines listed in NEMLIT were anti-infectives including antibacterials, antivirals, antimycobacterials and antimycotics. This means that most of anti-infective medicines have been distributed at all levels in the health care facilities^41^. Considering its usage at all level and if their enantiomers are either inactive or ineffective, may contribute to the occurrence of antimicrobial resistance. Consequently, if their quality with regards to enantiomeric purity is not known then the risk to the population will be higher due to potential existence of inactive, active or toxic enantiomers. Subsequently, there is a need to conduct enantiomeric purity analysis for anti-infective medicines circulating on the market. In addition, further studies should be conducted to investigate if the inactive or ineffective enantiomer is among contributing factor in the development of antibiotic resistance.

Moreover, these results indicate that, out of the top ten (10) commonly used chiral medicines, seven (7) were anti-infectives. In this study, two antibiotic chiral medicines were selected for future monitoring and linking their chirality with occurrence of adverse drug reactions. The first one is Amoxicillin (capsules and suspension) which ranks number one among the top ten and is most commonly used over all achiral and chiral registered medicines. The second one is ceftriaxone powder for injection which is number ten among the most commonly used chiral medicines. These two medicines have also been reported to be associated with adverse drug reactions as per UMC/WHO—Vigilyze database. It is not known if these adverse drug reactions are due to chirality of the medicines or not. A signal detection of adverse drug reaction identified by WHO—UMC reported a potential sub-group at risk which suggested that males may be at increased risk for drug induced aseptic meningitis with the use of amoxicillin and amoxicillin in combination with clavulanic acid^42^. Another 12 new signals were detected in Korea on the use of amoxicillin including ineffective amoxicillin^43^, however, the chirality factor was not reported to be associated with the new signals. In addition, a concern on safety of ceftriaxone has been reported for both adults and children^44,45^. Fatal adverse effects of injected ceftriaxone sodium were reported in China, the reason among others being the possibility of poor drug quality^45^. The results of our current study also call for more investigation to be conducted on safety and quality control of medicines especially anti-infective medicines, which are mostly used in the public. Investigations will help to determine enantiomeric purity against safety profile so as to establish if there is any relationship between chirality and reported adverse drug reactions. Enantiomeric chiral separation will prevent occurrence of any hazards to the public and may contribute to reduce risk of antimicrobial resistance in case of existence of inactive enantiomers.

Our study has limitations and strengths of which limitations are that, the study reviewed all medicines registered for the past 15 years from when TMDA (previously TFDA) was established (July, 2003). However, during the TMDA establishment time, the TMDA—Integrated Management Information System (IMIS) registration database was not in place and therefore retrieval of some data was conducted by using hardcopy documents in order to capture all information. One of the strengths is that, there was no missing data. Recorded data were verified and validated using common technical documents (CTD), Public Assessment Reports and search engines such as PubMed and Google scholar.

In conclusion, the study revealed the existence of both chiral and achiral registered human medicines in Tanzania with chiral medicines predominating. The study further revealed that, the proportion of racemates within chiral medicines group was significantly higher compared to single enantiomers. In addition, anti-infectives racemic mixtures were more registered and listed in NEMLIT than other pharmacological groups. The use of available medicines as racemates may cause harm to the public due to the potential existence of inactive and toxic enantiomers. Moreover, the use of racemic anti-infectives may also have consequences on antimicrobial resistance. In order to protect public health from any harm that might be caused by racemates, it is necessary for developing countries, Tanzania inclusive to develop chiral separation techniques especially during the quality control of these medicines. This is pivotal as chirality plays a key role in clinical management. There is also a need for regulatory bodies to strengthen the regulatory control of medicines to include determination of inactive and/or toxic enantiomers of chiral medicines during post marketing surveillance.

## Methods

### Study design

This was a retrospective cross-sectional study. All human medicines registered in Tanzania for the past 15 years from July 2003 to June 2018 were reviewed to ascertain their chirality status.

### Retrospective review of registered medicines

#### TMDA—IMIS registration database and Chirality review

We used the TMDA—IMIS registration database^26^ to obtain data source of all registered medicines for the past 15 years from July 2003 to June, 2018. The ratio and chirality types of registered medicines were determined by reviewing registered medicines listed in the registration database stationed at TMDA, Dar es Salaam, Tanzania. Veterinary medicines were excluded and only human medicines were studied. All human medicines that were withdrawn from the market and those which had no valid registration status were not included in the review.

In this study, all human medicines with valid registration were reviewed to identify their chirality. During the review, all information pertaining to brand name, generic name, dosage form, strength, ATC classification system (code), ATC description, manufacturer’s name and country of origin were exported to the Microsoft excel data sheet for analysis. Different tools such as registration dossier (common technical document—CTD) which consisted of technical information of the medicines submitted from manufacturers during the application for registration of medicines, TMDA medicines assessment reports; Public Assessment Reports published by Stringent Drug Regulatory Authorities (SDRA) and search engines such as PubMed and Google scholar were used. Data were cleaned, verified and validated to minimize entry errors and missing information. The ATC classification was used during the review of chiral medicines in order to assess their therapeutic classification based on their pharmacological group. Moreover, the review was extended to assess if these registered chiral medicines are among the listed essential medicines and the identification of commonly used chiral medicines were done by reviewing the TMDA importation database.

### ATC classification

ATC Classification is an internationally accepted classification system for medicines that is maintained by the World Health Organization (WHO)^46^. ATC codes have been assigned to all active substances contained in medicines based on the therapeutic indication. In the ATC classification system, the active substances were divided into 14 different groups according to the system on which they act and their therapeutic, pharmacological and chemical properties^46^.

Medicines were classified in five (5) different levels and divided into fourteen main groups (1st level), with pharmacological/therapeutic subgroups (2nd level). The 3rd and 4th levels are chemical/pharmacological/therapeutic subgroups and the 5th level is the chemical substance^46^. The 2nd, 3rd and 4th levels are often used to identify pharmacological subgroups and are considered to be more appropriate than therapeutic or chemical subgroups^46^. In this study, two levels “i.e.” 1^st^ and 2^nd^ were considered to comprehensively categorize registered chiral medicines into their pharmacological subgroups.

### NEMLIT

Chiral medicines were further reviewed to assess their existence in the NEMLIT to determine if they were among essential medicines. NEMLIT reflects the policy of the Government of Tanzania of ensuring availability of safe and efficacious essential medicines to all its citizens^41^. It is therefore, a key tool which is used effectively to promote access to essential medicines to achieve maximum therapeutic benefit and optimize patient outcomes. The NEMLIT fifth edition (2017) was used during the review. It is in line with the WHO model list of Essential Medicines (EML) 20th edition (2017). NEMLIT gives guidance to healthcare workers at all levels including dispensaries and health centers on the medicines to be prescribed. It also gives restrictions on the use of antibiotics in health facilities to those selected as the most appropriate for use at each level of health care delivery^41^.

### TMDA—IMIS importation database and WHO VigiLyze database

The most commonly used chiral medicines in Tanzania were assessed by using the importation database located at TMDA. All medicines imported for public and private use were assessed. In addition, the WHO VigiLyze database was also used to identify commonly used chiral medicines with reported adverse drug reactions.

### Data management and statistical analysis

The obtained data from the reviews were checked for any inconsistencies. The data was entered into a Microsoft Excel spreadsheet (version 2010), verified and exported to STATA Version 15 software for statistical analysis. Descriptive statistics was determined for types of chirality, ATC classification—pharmacological subgroup and those listed in NEMLIT. Moreover, countries of origin of chiral medicines registered in Tanzania and two mostly used chiral medicines in Tanzania were also identified. A one-sample t-test between proportions was performed to determine whether there was a significant difference between the percent of chirality type, country of origin, ATC classification and existence of chiral medicines in NEMLIT. The level of confidence required for significance was set at *p* < 0.05.

### Ethical consideration

Ethical clearance was granted by the Medical Research Coordinating Committee (MRCC) of the National Institute for Medical Research, Tanzania (Certificate No. NIMR/HQ/R.8a/Vol.IX/3086) and also was issued by Institutional Review Board of Muhimbili University of Health and Allied Sciences (MUHAS) certificate number DA.282/298/01.C.

## Data Availability

Dataset generated and analyzed during the study are available at TMDA office and TMDA website- registration of medicines ^26^. Detailed dataset for the previous 15 years (July 2003—June 2018) are available with permission of Tanzania Medicines and Medical Devices Authority (TMDA).
